# Nonsteroidal anti‐inflammatory drugs and pharyngitis

**DOI:** 10.1002/iid3.738

**Published:** 2022-11-25

**Authors:** Stuart B Mazzone, Anuradha Kulasekaran, Tim Shea, Oluwajoba Adegoke

**Affiliations:** ^1^ Department of Anatomy and Physiology, School of Biomedical Sciences The University of Melbourne Melbourne Victoria Australia; ^2^ Global Medical Affairs – Respiratory, Reckitt Slough UK; ^3^ Global Medical Affairs – Respiratory, RB Health (US) LLC Parsippany New Jersey USA; ^4^ Global Medical Affairs – Respiratory, Reckitt Hull UK

We read with interest Leyva‐Grado et al., “A novel anti‐inflammatory treatment for bradykinin‐induced sore throat or pharyngitis.”^1^ Clinical studies have shown bradykinin induces sore throat in healthy subjects^2^, ^3^ and the authors propose anti‐inflammatory treatments, in this case, aspirin should prevent bradykinin‐induced inflammation and relieve pharyngitis. However, features of the experimental design used to advance understanding of this mechanism, including the model system, choice of comparator products, and dosing and dissolution strategies warrant scrutiny. In our opinion, the interpretation of the results from this experimental data is insufficient to validate the aspirin formulation as superior in clinical efficacy for sore throat pain relief.

Leyva‐Grado et al.^1^ showed bradykinin applied to cultured airway cells resulted in a 2‐ to 4‐fold increase in PGE2 in the culture media, an effect inhibited by acetyl salicylic acid (aspirin)^1^ consistent with a cyclooxygenase‐dependent mechanism. However, it is difficult to know whether a 2‐ to 4‐fold change in PGE2 is above the threshold for any biological relevance. Care must be taken when extrapolating this finding to the clinical setting as modulation of inflammatory mediators in a culture system does not directly support the clinical efficacy of the treatment. Thus, whilst the study adds to substantial literature demonstrating anti‐inflammatory actions of aspirin, it falls short of showing clinical superiority in pharyngitis, despite the publication title claims of a “novel” “treatment” for “sore‐throat.”

The use of NSAIDs, such as ibuprofen, diclofenac, flurbiprofen, aspirin, in pharyngitis is common, effective, and recommended.^4^, ^5^ The authors employed an ambitious translational experiment to compare their new Biovanta aspirin products for pharyngitis to existing over‐the‐counter (OTC) products by applying each product directly to cultured alveolar pneumocytes or nasal epithelium grown at air–liquid interface.^1^ Notably, these cells develop into tissues akin to tracheobronchial pseudostratified columnar ciliated mucosa. The tissues may resemble the nasopharynx, but not the non‐keratinized stratified squamous mucosa of the oropharynx or laryngopharynx. This may seem a minor point when studying the fundamentals of bradykinin‐evoked mediator release. However, OTC products formulated for oral administration were directly applied to these ciliated epithelial cultures to assess product efficacy and toxicity superiority.^1^ Unsurprisingly, most products caused significant damage to these ciliated tissues which are not biologically “designed” to experience orally administered treatment formulations that contain preservatives and nontherapeutic compounds for palatability and physical characteristics.

Mechanistic drug studies routinely assess efficacy relative to other known modulators of the targeted pathway. Predictable results occur when inappropriate comparators are used. Leyva‐Grado et al.^1^ compared the efficacy of aspirin in Biovanta products either to marketed products containing active pharmaceutical ingredients (APIs) with no known anti‐inflammatory activity, including dextromethorphan (cough suppressant), guaifenesin (expectorant), and phenylephrine (decongestant), or to products devoid of true APIs. This design introduced a comparator bias, increasing the likelihood of demonstrating the superiority of the aspirin‐based Biovanta products. Furthermore, although liquid formulations could be diluted and applied to cell cultures, lozenges required dissolution using “saliva buffer.”^1^ However, the approach was not cohesive, as each lozenge was treated with the minimum volume of buffer needed to dissolve the predominant sugar in the lozenge based on sugar solubility and density. Considering, for example, that Cepacol Instamax lozenges are sugar‐free containing sugar alcohols such as maltitol and isomalt, it may have been appropriate to account for the lozenge weight to calculate buffer dissolution volumes. There is no publicly available information on the exact amount of sugar in many of the products tested and the design did not account for the large amounts of saliva produced while sucking a lozenge. Notably, the method employed resulted in the Biovanta lozenge having the highest dilution and co‐incidentally the lowest toxicity of the products tested.^1^

## 1 CONCLUSION

Understanding mechanisms causing pharyngeal inflammation and pain is important for developing new therapies to treat pharyngitis. Leyva‐Grado et al.^1^ support PGE2 as a mediator released from bradykinin‐stimulated airway epithelia via an NSAID inhibitable pathway. However, in our opinion, the interpretation of their results neither supports the superiority nor the clinical efficacy of the new aspirin products tested. Whether the Biovanta spray or lozenge proves better than placebo or other NSAIDs in treating sore throat awaits the outcomes of an adequately controlled clinical trial.

## CONFLICTS OF INTEREST

Stuart Mazzone has received funding from Reckitt Health Ltd. (Reckitt). All other authors are employees of Reckitt.
